# Legal aspects of newborn screening

**DOI:** 10.1515/medgen-2022-2110

**Published:** 2022-05-07

**Authors:** Henning Rosenau, Felicia Steffen

**Affiliations:** Chair for Criminal Law, Criminal Procedure and Medical Law, Law Faculty, Martin-Luther-University Halle-Wittenberg, Halle an der Saale, Germany

**Keywords:** newborn screening, Genetic Diagnostics Act, informed consent, incidental findings

## Abstract

Newborn screening is used for the early detection of diseases in newborns and enables rapid intervention to prevent serious consequences, including infant death. Since the Genetic Diagnostics Act came into force in 2010, the rules of the Act have applied to newborn screening. Over the years since the Act came into force, some legal issues have been resolved, but new legal aspects have also arisen for which the Act does not yet provide a solution.

## Introduction

Newborn screening is a procedure for the early detection and prevention of certain congenital metabolic and hormonal diseases. A blood sample is taken from the newborn’s heel during the first few days of life, the blood is placed in several drops on a test strip made of filter paper, and the test card is sent to a screening laboratory for analysis. The diseases, if left untreated, and especially without a rapid therapeutic response, can lead to mental or physical disabilities or even death. Early diagnosis allows the newborn to receive early treatment and prevent secondary damage. [1, p. 214] Newborn screening has therefore been an integral part of the health care system’s preventive examinations since the 1960s and 1970s. [2, p. 75]

The Genetic Diagnostics Act (GenDG – Gesetz über genetische Untersuchungen beim Menschen, July 31, 2009, BGBl. I 2009, 2529) has been in force in Germany since February 1, 2010. Newborn screening as genetic screening according to sect. 16 of the GenDG is subject to the Genetic Testing Act (see below, Newborn screening as an application of the GenDG section), so that sect. 8–10 of the GenDG apply to informed consent and, if necessary, genetic counseling. Implementing provisions can be found in the guideline “Genetic Screening” [3] of the Commission on Genetic Diagnostics (GEKO). This commission is a body of experts established at the Robert Koch Institute (RKI) and is mandated, among other things, by sect. 23 para. 2 of the GenDG to issue guidelines with regard to the generally recognized state of the art in science and technology. In addition, under sect. 23 para. 3 of the GenDG, it issues opinions under sect. 16 para. 2 of the GenDG on genetic screening with regard to its medical and ethical acceptability. It also addresses current issues relating to genetic testing in communications.

In addition to the GEKO, the legislature has assigned regulatory tasks for newborn screening to the Federal Joint Committee (G-BA). As a supreme decision-making body of the joint self-government in the German health care system, the G-BA has the task of specifying the catalog of services provided by the health insurance funds in accordance with the generally recognized state of medical knowledge. [4] The G-BA reports to the Federal Ministry of Health. The tasks and requirements of the G-BA are defined in the SGB V (Sozialgesetzbuch Fünftes Buch – Gesetzliche Krankenversicherung, December 12, 1988, BGBl. I 2477, 2482). The main task of the G-BA is to draw up guidelines to ensure the provision of medical care, sect. 92 para. 1 of the SGB V. For newborn screening, the Children’s Guideline [5] and the Maternity Guidelines [6] are authoritative.

## Applicability of the GenDG to genetic screening (Reihenuntersuchung)

### Newborn screening as an application of the GenDG

That newborn screening falls within the scope of the GenDG is now generally accepted. However, this was not self-evident when the new law was introduced. The first question was whether the already established newborn screening is covered as an application of the GenDG. Already the emphasis on “*gene*” – genetic *screening* – raised doubts. The vast majority of the diseases covered by newborn screening are actually genetic, but not all. This concerns, for example, the most frequently recorded disease, hypothyroidism. [2, p. 75] Furthermore, the aim is not to identify a genetic predisposition or predisposition carrier, but to diagnose a disease for which a necessary therapy can be initiated. [2, p. 75] It is also conceivable that future new target diseases will not necessarily be genetic. The definition formulated in the GenDG for genetic screening cannot be readily applied to newborn screening.

According to sect. 3 no. 9 of the GenDG, a “genetic screening is a genetic examination for medical purposes that is systematically offered to the entire population or to certain groups of persons in the entire population without there necessarily being any reason to assume that the respective person concerned has the genetic characteristic whose presence is to be clarified by the examination.”

While newborn screening is an offer to a specific group of people in the population as a whole (the newborn), it is not always, as stated, a *genetic* trait that is clarified by the screening.

In addition, it is difficult to draw the line between a genetic *screening* and a simple genetic examination. The latter requires an individual indication for carrying out the examination. The focus is on the individual motivation and the individual benefit of the individual. In contrast, genetic screening can also be carried out “without there necessarily being any reason to believe that the person concerned has the genetic characteristics”, sect. 3 no. 9 of the GenDG. This is justified by a general public interest in a systematic offer. The differentiation according to the motivation is not entirely clear. Why should not every newborn also have an individual benefit to participate in screening? The screening is offered precisely because it is intended to exclude for the newborns that there is an increased probability of the presence of a previously unnoticed disease, because it has not yet broken out in one of the parents. Parents thus participate precisely with the individual motivation to protect their child from dangerous, sometimes fatal, diseases. Moreover, screening is offered because there is an increased risk of disease in the population of newborn children. [2, p. 81; other opinion 7, p. 213]

Finally, also the question arises as to when the offer is made “*systematically* to the entire population or to specific groups of persons in the entire population”, sect. 3 no. 9 of the GenDG, meaning that it has a “program character”. [3, II.1.] An offer has program character when, according to “clearly defined and binding procedures, all persons in the target population” [3, II.1.] are addressed and examined with their consent. But how specifically must this predefined target population be named? Is it already sufficient for a systematic offer if “all women over 50” are invited to a screening? Such a prevention offer, which is generally directed at such a group, will not be sufficient. In addition, there must be a targeted approach to the members of such a population, so that, legally speaking, it is not merely an invitatio ad offerendum, but a clearly targeted, basically binding offerendum ad incertas personas. The guidelines of the GEKO speak of proactive approach. [3, II.1.] This is the case with newborn screening. They or their parents are specifically and directly selected, and they are systematically made a clear, unambiguous, and binding offer to participate.

The problem of classifying newborn screening as a genetic screening was resolved by the fact that the legislator explicitly mentions newborn screening as an example of a serial genetic examination in the explanatory memorandum to sect. 16 para. 2 of the GenDG. [8, p. 33] In its first activity report, the GEKO also confirmed that newborn screening is a genetic screening. [9, p. 16] The requirement for a statement by the GEKO in accordance with sect. 16 para. 2 of the GenDG was also not necessary for the already established newborn screening, because this provision only applies to those genetic screening tests that are introduced after the law comes into force and are therefore only started then. [8, p. 33]

However, prior review and evaluation by the GEKO is required for the *expansion* of newborn screening to include new target diseases. The legislature has deliberately opted for a restrictive handling of screening offers because, viewed as a whole, it is not so much the individual interest that matters, but primarily the general interest. [2, p. 80] The public interest and the state’s duty of care are the reasons why the GEKO recommendation for genetic screening is required. [3, IV.] When the public interest is placed before the individual interest of the individual, control duties fall on the state. [8, p. 33] The performance of genetic screening and thus the assumption of a public interest can only be justified if new target diseases are checked against the requirements of sect. 16 para. 1 of the GenDG. Above all, it must be clarified whether the target disease is treatable and thus whether a benefit for the persons concerned can be demonstrated. [3, II.1.] Should an evaluation by the GEKO not be necessary in the case of an addition to newborn screening, the requirements of sect. 16 of the GenDG would no longer play a role. This is not compatible with the purpose of sect. 16 para. 2 of the GenDG. In contrast to the already established newborn screening, there is also no legitimate expectation that screening will continue as before. Therefore, in the event of significant changes and extensions to the newborn screening, the evaluation must be carried out by the GEKO. [2, p. 80] Since the GenDG came into force, newborn screening for five new target diseases has already been included after positive evaluation by the GEKO: cystic fibrosis, tyrosinemia type I, severe combined immunodeficiencies (SCID), 5q-associated spinal muscular atrophy (SMA), and sickle cell disease (SCD). [10]

### New genetic screenings?

Apart from newborn screening, no other genetic screening tests have been established so far. However, there are already a number of preventive examinations offered that are not (yet) designated as genetic screening, for example, prenatal screening, which is generally offered to women over 35 [2, p. 81], and the established mammography screening. The latter for example is an X-ray examination and therefore not a *genetic* screening. In other cases, the genetic aspect might already be clear, but it would have to be a genetic screening designed as a *systematic* offer. The demarcation between one form and another is – as described – not very clear. [11, p. 75] The following borderline cases show the difficulties in applying sect. 16 of the GenDG.

#### Non-invasive prenatal diagnostics for the determination of the fetal rhesus factor within the framework of the Maternity Guidelines (Mu-RL)

In August 2020, the G-BA included non-invasive prenatal diagnostics (NIPD) for the determination of the fetal rhesus factor in the Mu-RL. [12] This non-invasive prenatal diagnostic is a genetic analysis within the framework of a prenatal genetic examination, sect. 3 no. 1 lit. a of the GenDG. [13]

According to the Mu-RL, every RhD-negative pregnant woman with a singleton pregnancy should be offered the determination of the fetal Rh factor on fetal DNA from maternal blood. [12, modification of chapter C by integrating No. 2] Accordingly, the offer is directed to all pregnant women with the Rh factor negative, i. e., a specific population group. However, the assignment requires that the rhesus factor is determined in a pregnant woman. This medical finding is therefore only available after a specific examination. It is only on the basis of this preliminary examination that non-invasive prenatal diagnostics to determine the fetal rhesus factor takes place.

However, a genetic screening presupposes a “proactive approach to a specific group of persons or to the entire population, without the selection of the group of persons being based on prior medical findings.” [3, II.2.] Thus, the selection of RhD pregnant women on the basis of a prior medical finding argues against the classification as genetic screening. Rather, the genetic analysis to determine genetic characteristics is to be performed precisely because of the Rh factor found in the mother.

For the same reason, non-invasive prenatal diagnostics to determine the risk of autosomal trisomies 13, 18, and 21 [14] does not constitute genetic screening, because the offer of screening is associated with the prior determination of the present medical or psychological indication in the pregnant woman. [3, II.1.]

#### “Vroni study” in Bavaria

Other borderline cases are newly initiated studies, such as the “Vroni study.” This is a Bavaria-wide screening for “familial hypercholesterolemia” (FH). [15] This is an inherited metabolic disorder that is associated with a significant increase in cholesterol in the blood. This can build up in the vessels and clog them, which can lead to a heart attack. The study offers all children in Bavaria between the ages of 5 and 14 free early detection of the disease. To conduct the study, the child’s blood is first drawn and the cholesterol level is determined. Genetic diagnostics is only performed if cholesterol levels in the blood are found to be much too high. [16] Similar to the considerations regarding the classification of NIPD for the determination of the rhesus factor, this could already speak against the classification as a screening, because the blood is first examined and there is thus a preliminary finding.

It could also be questionable whether the study, in its capacity as a study, falls within the scope of the GenDG at all. Genetic examinations for research purposes are excluded, sect. 2 para. 2 no. 1 GenDG. The “Vroni study” is a sub-project of “DigiMed Bayern”, in which various “research activities” are involved. [17] The DigiMed Bayern project has a duration of five years and a number of funding partners. [18], [19] The fact that the “Vroni study” is part of this project indicates that the focus is on research. [20, p. 5 detailed on this criterion] In this case, the GenDG does not apply.

## Informed consent in the context of newborn screening

### Information and physician’s reservation

As a case of application of the GenDG, the general regulations of its 2nd chapter on informed consent and genetic counseling according to sect. 8–10 of the GenDG apply to newborn screening. Likewise, the physician’s reservation in sect. 7 of the GenDG applies, according to which the information may only be provided by the responsible medical person. Since this is a diagnostic genetic examination and not a predictive question, the simple physician’s reservation of sect. 7 para. 1 Alt. 1 of the GenDG applies: the information must be provided by a physician.

However, the practical application of such a clear regulation proves to be difficult. The physician’s reservation precludes midwives and maternity nurses from performing newborn screening. [21, sect. 7 No. 6; 22, sect. 7 No. 11] However, these are the only ones with whom the pregnant woman regularly meets in childbirth. Where a physician is not present at the birth, lack of medical information could mean that newborn screening cannot be performed. [22, sect. 7 No. 11]

In its pediatric guideline, the G-BA aimed for a practicable solution and circumvented the legal regulation: If the birth is conducted by a midwife or a delivery nurse, “the information can be provided by them if the possibility of consultation with a physician is guaranteed.” [5, Annex 2 sect. 4 para. 1 sent. 2] This regulation has also been accepted by the GEKO and supported in its updated guideline “Aufklärung medizinische Zwecke”. [23, II.6.] In the awareness that in the strict application of the GenDG an informed consent may not be obtained and thus the newborn screening may not be performed and then the newborn may be seriously harmed, this is a pragmatic solution. [2, p. 79] Therefore, this procedure is to be tolerated and accepted, even if the regulation is difficult to reconcile with the GenDG. The physician’s reservation is clearly regulated there. According to the legal regulatory model, delegation of the informed consent would only be possible to other physicians and only under certain supervision by the responsible physician. [2, p. 79]

The current model is not a satisfactory solution in the long term. Instead, the process should be adapted. In the future, newborn screening could be discussed with physicians during the initial interview with the pregnant woman before the birth. A corresponding regulation could be included in the G-BA maternity guidelines, whereby at the same time the remuneration of the information would have to be regulated.

### Parents’ information on extended newborn screening

The G-BA’s Children’s Guideline regulates the content of the age-related examinations that take place in infancy and childhood immediately after birth until the age of 6. It also specifies the details of early detection examinations such as extended newborn screening. [5] Annex 3 of the Children’s Guideline contains parent information on the extended newborn screening. Parents can obtain an overview of the procedure and the diseases examined via the G-BA’s parent information on extended newborn screening. With regard to the diseases examined, parents are informed that in most of the families concerned, no such diseases have yet been present: “No statements about family risks can be derived from this examination alone.” [5, Annex 3]

However, this formulation is scientifically incorrect in the way it is formulated. The diseases for which newborn screening is carried out may well be genetic and then allow conclusions to be drawn. This applies, for example, to tyrosinemia type 1 and SCID. This was clarified by the GEKO in its statement on the screening of newborns for the early detection of SCID [24] in accordance with sect. 16 para. 2 of the GenDG after reviewing and evaluating the information provided to parents. The information provided prior to genetic testing, the contents of which are governed by sect. 9 of the GenDG, requires that the parental information also specify “the important and necessary information that SCID, as well as most of the investigated target diseases of expanded newborn screening, are genetically determined”. [24] Therefore, a change in the current wording is warranted (this is probably in preparation), precisely because most of the disease screened for is hereditary, i. e., genetic. Although it is true that such examinations alone “do not *usually* allow any conclusions to be drawn about familial *predispositions*” [24, emphasis by the authors], in individual cases they can. This must not be withheld from the parents. Only the ill-founded fear that parents will then be deterred from taking part in the screening – because there is talk of too much genetics – does not justify any compromise on informed consent.

At the same time, the GEKO also pointed out in its statement that, in the context of the information provided pursuant to sect. 9 para. 2 no. 1 of the GenDG, information must be provided on all medically relevant results that can be achieved with the investigational device used. [24] There would therefore have to be a clearer formulation for newly included target diseases so that possible incidental findings could be collected. This is a fundamental problem of any genetic examination, touching on the antagonistic rights of not to know and to know and encountering a partly dysfunctional regulation in the GenDG [25, p. 437 f.], not a specific problem of newborn screening. Regarding the latter, the GEKO writes: “Conspicuous results in SCID screening can also provide indications of other genetic and non-genetic diseases. Children affected by these may also benefit from therapy.” [24]

The implementation of these proposals has so far been rejected by the G-BA in its supporting reasons for the G-BA’s decision on the amendments to the Children’s Guideline. [26] The information for parents would indicate that the GEKO deals with genetic diseases. [26, Annex 3, p. 15] In the same way, the information of the parents with regard to possible side findings is part of the medical information. [26, Annex 3, p. 15] The G-BA opposes a reformulation or an addition because it is of the opinion that it would jeopardize the comprehensible presentation of the information for parents. [26, Annex 3, p. 12, 15] Here, the objection just expressed applies cum grano salis accordingly. The objections are neither substantiated nor can they be upheld against the background of a comprehensive informed consent concept, which is reflected not only in medical law in sections 630c and 630e of the German Civil Code (BGB), but also in sect. 7 of the GenDG in particular.

## Handling of the obtained results

Sect. 11 of the GenDG regulates how the results of a genetic examination are to be handled and to whom and by whom the findings are to be disclosed. Sect. 11 of the GenDG serves the patient’s right to informational self-determination. [27, Sect. 11 No. 1] In addition, the provision safeguards the protection of the physician’s prerogative for genetic testing as determined by the legislator and the associated communication with the person concerned by preventing the patient from being confronted with his or her results outside the physician-patient relationship. [27, Sect. 11 No. 1; 2, p. 85]

### Communication of results

According to sect. 11 para. 1 of the GenDG, a result of a genetic examination may only be communicated to the person concerned and only by the responsible physician. Sect. 11 para. 2 of the GenDG safeguards the legislative concept by stating that the laboratories or laboratory physicians conducting the tests are not authorized to communicate the results of the analysis to the patient or a third party. Only the responsible physician who commissioned the analysis may be sent the results.

In everyday practice, the implementation of these requirements can prove difficult. Even in clinics or large practices, where procedures are regularly characterized by shift work, a temporary absence of the responsible medical person is unavoidable. [27, sect. 11 No. 2c; 2, p. 84] However, on the part of the person concerned, in the case of newborn screening the parents, there is an interest in the results being transmitted without delay. [27, sect. 11 No. 2c] Otherwise, waiting could result in harm to the newborn if necessary therapeutic measures are not initiated immediately. [27, sect. 11 No. 2c; 2, p. 85]

The GEKO had therefore already expressed in its 5th announcement [28] on the proxy rule for notification of results that a broad interpretation of the wording of the law may be appropriate. “In rare emergencies, where there is a risk to the life or physical integrity of the patient and timely notification of results cannot be made by the persons appointed to do so, the results may also be notified to the patient by other persons.” [28] Presumed consent is to be assumed. In these cases, the above-mentioned laboratories may also communicate the test results to other physicians.

This option is likely to be chosen not infrequently for newborn screening as well. [27, sect. 11 No. 2b] In this case, rapid action is required to avoid damage to the child’s health. [2, p. 84] The G-BA Children’s Guideline stipulates that no more than 72 hours may elapse between sample collection and the transmission of an abnormal finding. [5, sect. 18 para. 4]

It therefore appears dysfunctional when the G-BA, in its decision to amend the Children’s Guideline of August 20, 2015, when including the new target disease cystic fibrosis [29] in sect. 37 para. 2, stipulates that the notification of the result of the DNA analysis from the screening laboratory to the attending physician must be made via the physician who initiated the genetic screening. This will usually be the physician at the maternity clinic. [27, sect. 11 No. 2b] Forwarding of results may be necessary if a test does not provide clear results and the DNA analysis results already available can be used in confirmation diagnostics. [30, p. 18]

This regulation of sect. 37 para. 2 is a considerable logistical complication for the confirmed diagnosis of cystic fibrosis. [30, p. 17] Initially, the G-BA had also regulated the transmission of findings in its draft of the Children’s Guideline in such a way that the screening laboratory could transmit the results directly to the pediatrician performing the confirmation diagnosis. [30, p. 17] However, the G-BA then moved away from this and changed the regulation to the effect that the transmission of results from the screening laboratory must go via the responsible medical person who initiates the screening and is therefore also responsible (usually the maternity clinic). Direct – and this is what is important – rapid transmission from the screening laboratory to the attending pediatrician is not possible. This detour is not necessary and may be highly disadvantageous for the newborn. The parents’ wishes must be complied with and, if parental consent is given, the result must be transmitted as quickly as possible. This is also expedient because only the laboratory has the results of the DNA analyses. [30, p. 18]

This method of communicating the results is also compatible with the GenDG. Sect. 11 para. 2 of the GenDG does not exclude the possibility of consent, either from its wording or from the intention of the legislator. [2, p. 85; other, not convincing opinion 7, p. 272 f.] Therefore, this dangerous detour is also not legally necessary. Direct transmission is appropriate in particular for the protection of life and physical integrity. The right of self-determination is safeguarded by the parents’ consent to direct transmission of the result. It also excludes the possibility of the physician initiating the test finding out about the result before it is passed on. [30, p. 18; 22, II.4] In accordance with the view of the GEKO, the Federal Ministry of Health – to which the G-BA is subordinate – has also asked the G-BA “to examine, at the latest within the framework of the evaluation according to sect. 42, how the method of notification of findings provided for in sect. 37 para. 2 of the revised version of the Children’s Guideline is implemented in practice, whether problems have arisen in its practical implementation, as well whether and to what extent there have been negative effects on the quality assurance procedure concerning screening for cystic fibrosis and whether the regulation thus – in the result – requires an adjustment”. [31] The Federal Ministry also points out that it considers the path initially envisaged by the G-BA, as it was based on the opinion of the GEKO, to be compatible with the provisions of the GenDG.

There is reason to hope that the G-BA will adjust the regulation when the Children’s Guideline is amended.

### Incidental findings

Another topic concerns the handling of results that were collected during newborn screening but do not relate to any of the target diseases of newborn screening and were therefore not collected with the aim of being able to initiate preventive measures. These results may nevertheless be of significance for the newborn or also for genetic relatives.

Also in classical diagnostics, the physician is confronted with findings that are not related to the actual diagnosis. For such “incidental” findings, such as the indication of a tumor in the lung on an X-ray taken by an anesthesiologist in preparation for an operation, the Federal Court of Justice (BGH) has laid down guidelines. [32] The physician has a duty of care, which requires him or her to carefully evaluate an X-ray and to assess “pathological finding(s) or at least a finding(s) requiring control”. [32, p. 31] This is because a pathological finding can be countered with preventive measures. It is of such relevance that a duty to act arises from the physician’s care. Accordingly, a physician must check whether the – incidental – finding that has become visible is pathological and possibly in need of treatment. If there is a need for treatment, the physician must investigate the abnormality, even if it is not related to the actual objective of the examination: “He may not close his eyes to ‘incidental findings’ that are recognizable to him in this sense”. [32, p. 35] This was confirmed by the Federal Court of Justice in a further ruling of 26.5.2020 [33], according to which the doctor must follow up on indications of a serious illness.

The handling of surplus information – incidental or random findings – in the context of genetic diagnostic examinations is the subject of lively debate and is largely unresolved. For example, it is uncertain to what extent the assessment of the Federal Court of Justice can be transferred to the GenDG and has significance for genetic screening. In the final analysis, this will have to be rejected. This is because the law is based on the idea of genetic exceptionalism, i. e., the assumption that genetic health data have a special position because they are relevant over long periods of time with their personal identity reference. These health data are associated with high predictive potential, possibly revealing information about third parties. [8, p. 1] Considerations that arise for the clarification of incidental findings in conventional medicine can therefore hardly be transferred one-to-one to the genetic context. [34, p. 110 f.; other opinion 11, p. 337 f.; 35, p. 402 even if there is severe suffering]

Another problem: in the context of newborn screening, a heterozygote result could represent such an incidental finding. In newborn screening, it may actually only be a matter of detecting such a disease, which can break out in the examined person himself. [27, sect. 16 No. 2] Thus, it is not about heterozygote screening, in which predispositions are to be detected whose changes only manifest themselves in the offspring. [27, sect. 16 No. 2] The aim is to identify a genetic predisposition to diseases and health disorders in the persons examined themselves, for which preventive measures can be initiated as soon as possible.

Despite this objective, it cannot be ruled out that the screening may reveal an indication of a corresponding investment carrier as a secondary finding. Such findings will no longer be an exception in the future. [34, p. 100] The heterozygote result can have a benefit for the child itself as well as for its genetic relatives. The predisposition can have an influence on the later reproduction of the newborn. Likewise, it may have an influence on the further family planning of the parents who plan to become pregnant again. From the heterozygote status of the child, a corresponding status of a parent could be inferred in whom the predisposition has not (yet) manifested itself. In case of a new pregnancy, another child could be affected. In this respect, it is clear what significance a notification of results can have for the parents at the time of the findings and for the child in adulthood. Especially in the field of genetics, the results regularly also have “multidimensional components.” [36, p. 74] So should the parents be informed about the result? The GenDG does not offer a solution to this conflict.

First of all, only treatable diseases may be collected and communicated as part of newborn screening because they have a direct benefit for the newborn. The legal idea can be taken from sect. 14 para. 1 no. 1 of the GenDG, which provides for a corresponding regulation in the case of medically indicated genetic examinations of persons who are not capable of giving consent. This must then apply a fortiori to genetic screenings that are not medically indicated in individual cases. Only a direct benefit justifies the restriction of the right of self-determination of the person concerned, where the right to know and not to know of the newborn child takes a back seat. [3, II.1.] The heterozygote result is not covered by this.

Therefore, with regard to the newborn, a clinically irrelevant heterozygote status should only be communicated to the newborns upon request in the course of genetic counseling if the capacity to consent exists. [37, VII.3.] Then, however, there must also be a mechanism for safeguarding results to ensure that this later notification is implemented. For this purpose, data storage would have to exist. However, the GenDG stipulates that results are only stored for ten years, sect. 12 para. 1 of the GenDG. Longer storage can be requested, sect. 12 para. 1 sent. 2 of the GenDG. However, if the person concerned, in this case the newborn, or the parents do not see any evidence that this would be necessary, there is no reason for them to agree to longer storage. Moreover, this additional question would considerably increase the amount of information required and would overburden a decision in the context of a “mere” serial examination. It would be a matter for the general public to organize longer backup of the data at a centralized location, which the then adult newborns can access if they wish.

With regard to the significance of the result for genetic relatives, i. e., an extraneous use, the conflict between the interests of the newborn and third parties cannot be resolved under the current legal situation. It is true that sect. 14 para. 2 of the GenDG stipulates that genetic may be carried out on persons who are incapable of giving consent in the case of third-party benefit. This third-party use of genetic testing refers precisely to the case described above in which a planned pregnancy is pending in genetically related persons. If, on the other hand, it is a matter of newborn screening, the genetic examination is carried out as part of a serial examination. There is no targeted examination for heterozygote status. The exceptional case of sect. 14 para. 2 of the GenDG cannot apply in the case of a serial examination. [7, p. 250]

Sect. 13 para. 2 of the GenDG, according to which the consent of the person concerned can be given for further use, cannot help either. Sect. 14 para. 3 of the GenDG, as a lex specialis, stipulates that only those examinations of the genetic sample that are necessary for the respective purpose of the examination are permissible. No other determinations may be made. This again clearly emphasizes the importance of the right to informational self-determination of persons who are not capable of giving consent. The opening clause of sect. 13 para. 2 of the GenDG therefore does not apply to persons who are not capable of giving consent.

With regard to sect. 11 of the GenDG, the legislator also accords greater importance to the investigated person’s right to informational self-determination than to the rights of relatives. [11, p. 289] In particular, the person being examined could also refuse to be informed in accordance with sect. 11 para. 4 of the GenDG, even though the result also affects relatives. It is only suggested in the context of genetic counseling that the person being examined also recommends counseling to relatives, sect. 10 para. 3 sent. 4 of the GenDG.

These findings are not satisfactory. The external benefit of the heterozygote result is obvious, in the case of the intrinsic benefit it is unclear whether the later communication to the newborn is ensured.

Conflicts also arise outside of newborn screening. For example, a person who wishes to check her risk of breast cancer, but for which genetic testing of a mother, who is unable to give her consent, is required, would not fall within the scope of sect. 14 of the GenDG. This is because the genetic examination would aim to determine a predisposition in the daughter but would not be linked to reproductive decisions.

The examples show that the extraneous benefits of genetic testing have not yet been sufficiently taken into account. It would make sense to consider possible exceptions to the general restriction. However, care must be taken to ensure that the special need for protection of persons who are incapable of giving consent is guaranteed as comprehensively as possible.

## Conclusion

Newborn screening has become a central area of modern genetic testing. The GEKO uses a large part of its deliberations on these issues. However, the legislature has only marginally considered newborn screening. The rules of the GenDG have been developed predominantly for a targeted, genetic testing of an individual patient for which there is concrete cause. Almost inevitably, this results in regulatory gaps and breaks in the application of the standard program of the GenDG to newborn screening. In the meantime, these have become obvious and sufficiently described, as also here. Therefore, it would now be time for the legislator to turn the issues into “maculature” with “three corrective words” [38, p. 23] and to ensure clarity.
